# Food addiction as a mediator in the association between depression and body mass index among young adults in South Korea: a cross-sectional study

**DOI:** 10.7586/jkbns.26.032

**Published:** 2026-08-25

**Authors:** Hyegyeong Cha, Seunghye Yun, Bokyoung Kim

**Affiliations:** 1 Department of Nursing, Namseoul University, Cheonan, Korea; 2 College of General Education, Namseoul University, Cheonan, Korea; 3 College of Nursing, Kyungpook National University, Daegu, Korea; 4 Research Institute of Nursing Innovation, Kyungpook National University, Daegu, Korea

**Keywords:** Body mass index, Depression, Food addiction, Obesity, Smell

## Abstract

**Purpose:**

This study examined the relationships among olfactory function, depression, food addiction, and body mass index (BMI) and assessed whether food addiction mediated the associations of olfactory function and depression with BMI among young Korean adults.

**Methods:**

This cross-sectional descriptive study included 158 adults recruited from a university campus in Cheonan, South Korea (mean age, 25.10 years). Olfactory function, depressive symptoms, and food addiction were assessed using the YSK olfactory function test, Patient Health Questionnaire-9, and Yale Food Addiction Scale 2.0, respectively; BMI was measured using bioelectrical impedance analysis. Data were analyzed using SPSS version 24.0 and the PROCESS macro version 4.2.

**Results:**

Current smoking status (β = .22, *p* = .006), depression (β = −.20, *p* = .024), and food addiction (β = .18, *p* = .044) were significantly associated with BMI. Food addiction significantly mediated the association between depression and BMI (B = 0.07, 95% bias-corrected bootstrap confidence interval: 0.01–0.15) but not the association between olfactory function and BMI. Depression had a positive indirect association with BMI through food addiction, whereas its direct association with BMI was negative, indicating an inconsistent mediation, or suppression, pattern.

**Conclusion:**

Food addiction mediated the relationship between depression and BMI in this sample. Depression was associated with higher BMI through addictive-like eating rather than through its direct pathway. Healthcare providers may consider screening for addictive-like eating behaviors as a modifiable target for obesity prevention among young adults with depressive symptoms.

## INTRODUCTION

The World Health Organization regards obesity as one of this century’s foremost public health challenges [1]. Obesity, characterized by excessive body fat accumulation, is commonly assessed using body mass index (BMI), calculated as body weight (kg) divided by height squared (m^2^) [2]. Globally, approximately one in eight people is obese, and 43% of adults are overweight [3]. In Korea, obesity prevalence has risen steadily across all adult age groups, particularly among those in their 20s, increasing from 22.4% in 2012 to 33.5% in 2024, a sharper rise than in any other age group [4]. As young adults are more susceptible to weight gain, they are a key target group for obesity prevention [5]. Although obesity has multifactorial causes, frequent consumption of energy-dense, ultra-processed foods rich in added sugars and saturated fat is associated with weight gain and increased obesity risk [6]. Such behaviors are influenced by both food availability and individual psychological and behavioral factors, with food addiction emerging as an important concept for explaining their contribution to obesity.

Food addiction is characterized by symptoms resembling substance addiction, such as impaired self-control, intense cravings, persistent eating despite negative consequences, and unsuccessful efforts to control food intake [7,8]. The Yale Food Addiction Scale 2.0 (YFAS 2.0) operationalizes this concept by adapting the 11 Diagnostic and Statistical Manual of Mental Disorders, Fifth Edition (DSM-5) criteria for substance use disorder, including loss of control, craving, tolerance, and withdrawal, to the overconsumption of highly processed foods [9]. Food-addicted individuals often experience strong cravings for energy-dense foods high in calories, sodium, and artificial flavorings. Like addictive substances, these foods stimulate the brain’s reward circuitry, potentially promoting compulsive eating behaviors [10]. A study of young American adults reported a food addiction prevalence of 21.9% [11]. Food addiction is associated with mood disorders, anxiety, other addictive behaviors [10], and obesity [12], suggesting it may represent an important mechanism linking psychological factors to obesity.

Olfactory function has also received attention as a potential obesity-related factor, given its crucial role in food selection, food intake, appetite regulation, and satiety [13–15]. Olfactory signals are transmitted to limbic and reward-related regions of the brain, creating a direct anatomical pathway through which food odors may elicit craving and hedonic responses [16,17]. Additionally, the olfactory bulb contains receptors for appetite-regulating hormones, which can alter olfactory sensitivity according to an individual’s metabolic state. This interaction forms a bidirectional olfactory–hypothalamic axis linking the sense of smell to central appetite control [16,18]. Consistent with this, appetite-regulating hormones have been found to modulate odor-induced activity in the human hypothalamus and olfactory cortices [17]. As odor cues can stimulate cravings through reward-related pathways while metabolic signals reciprocally reshape olfactory perception, changes in olfactory function could be associated with addictive-like eating and weight-related outcomes. However, this pathway remains inadequately established, and existing empirical evidence is mixed. Some studies have identified impaired olfactory function [15] and reduced olfactory sensitivity [19,20] in individuals with obesity, whereas others have observed increased sensitivity and greater preferences for specific food-related odors [21]. Obese individuals have also demonstrated stronger food-craving responses to food odors even when satiated [14,22,23] as well as enhanced hedonic responses to palatable odors [21]. Taken together, these findings suggest that the olfactory function–obesity relationship may involve addictive-like eating behaviors rather than olfactory sensitivity alone, providing a rationale for investigating food addiction as a potential mediator of this association.

Depression is a psychological factor associated with obesity, as affected individuals often use food to relieve negative emotions, potentially contributing to food addiction [11,24]. Furthermore, depression is associated with functional changes in neurotransmitter systems involved in appetite regulation, potentially promoting overeating [25]. Depression has also been linked to unhealthy dietary patterns contributing to weight gain [26], while the association between depression and obesity involves complex bidirectional biological interactions [27]. In line with these mechanisms, a previous study reported that food addiction symptoms fully mediated the association between depressive symptom severity and BMI [28].

Research has examined the individual associations of depression, food addiction, and olfactory function with BMI. However, studies simultaneously analyzing these variables within a unified framework or exploring the potential mediating role of food addiction in their associations with BMI remain limited. Earlier mediation studies demonstrated that food addiction mediates the relationship between depression and BMI in a general adult population [28] and between perceived stress and BMI among young adults [29]. Nevertheless, each of these studies focused on only a single psychological predictor, and neither included olfactory function as a physiological predictor. Therefore, this study aimed to investigate the relationships among olfactory function, depression, food addiction, and BMI in young Korean adults and examine the mediating effect of food addiction in the associations of olfactory function and depression with BMI. The findings may elucidate the pathways underlying BMI variations among young adults and offer insights for the development of weight-management interventions.

## METHODS

### 1. Study design

This study used a cross-sectional descriptive design to examine the relationships among olfactory function, depression, food addiction, and BMI in young Korean adults. Additionally, food addiction was examined as a potential mediator in the olfactory function–BMI and depression–BMI relationships. This study was reported in accordance with the Strengthening the Reporting of Observational Studies in Epidemiology (STROBE) reporting guideline for cross-sectional studies.

### 2. Participants

Adults aged 19 years or older were recruited from Namseoul University in South Korea, and no upper age limit was applied at enrollment. The study targeted young adults, defined as individuals aged 19 to 39 years in accordance with the age stratification applied in Korean national obesity statistics [30]. Eligibility criteria were the ability to understand the study’s purpose, to complete the questionnaires independently, and to provide written consent. Exclusion criteria included (1) olfactory dysfunction attributed to rhinitis, sinusitis, nasal polyps, or prior septoplasty; (2) a history of stroke, psychiatric disorders, hypothyroidism, or kidney or liver disease associated with anosmia or hyposmia; (3) olfactory impairment due to chemical exposure or medical procedures; and (4) inability to complete the assessments adequately due to cognitive impairment or other conditions. These exclusion criteria were assessed using self-reported responses to screening questions.

The required sample was determined with G*Power 3.1.9.7. The calculation was based on linear multiple regression using a fixed model, with an R^2^ increase, assuming a medium effect size of f^2^ = 0.15, a significance level (α) of .05, statistical power (1−β) of .95, five tested predictors, ten total predictors, and a two-tailed test. As previous studies did not provide comparable effect-size estimates, the medium effect size recommended for multiple regression analysis was adopted [31]. The minimum required sample was 138 participants. Anticipating about 20% attrition, 166 participants were recruited. After applying the exclusion criteria and removing participants with incomplete or unsuitable questionnaire or test responses, 158 participants remained for analysis.

### 3. Instruments

#### 1) BMI

Body weight and height were measured using an InBody 970 (InBody Co., Ltd., Seoul, Korea) and used to compute BMI. Based on the criteria of the Korean Society for the Study of Obesity, which are consistent with the Asia-Pacific cut-off values, participants were classified as having underweight (< 18.5 kg/m^2^), normal weight (18.5–22.9 kg/m^2^), overweight (23.0–24.9 kg/m^2^), or obese (≥ 25.0 kg/m^2^) [2].

#### 2) Olfactory function

Olfactory function was assessed with the YSK olfactory function (YOF) test (Kimex Co., Suwon, Korea) [32], which evaluates all three domains of olfactory function: threshold, discrimination, and identification. The olfactory identification component comprises 12 odorants, including 8 representing major functional groups and 4 familiar to Korean individuals. Scores from 12 trials each on the olfactory threshold, discrimination, and identification tests were summed to yield a total score of 36 points. Total scores were classified as follows: ≤ 15, anosmia; 16–21, hyposmia; and ≥ 22, normal olfactory function.

#### 3) Depression

Depression was assessed using the Patient Health Questionnaire-9. Kroenke et al. [33] developed the instrument, and An et al. [34] later developed and validated a Korean version. Its nine items correspond to the criteria for major depression and provide both a screening decision and a severity rating. Total scores of 0–4 indicate no depression; 5–9, mild depression; 10–14, moderate depression; 15–19, moderately severe depression; and 20–27, severe depression. A total score ≥ 10 is generally used as the cut-off for identifying clinically significant depressive symptoms [33,34]. Cronbach’s α was .95 in the study by An et al. [34] and .88 in this study.

#### 4) Food addiction

Food addiction was assessed using the YFAS 2.0, originally developed by Gearhardt et al. [9] and adapted into Korean and validated by Shin et al. [35]. The scale comprises 35 questions, including 33 items covering 11 diagnostic criteria derived from the DSM-5’s substance-related and addictive disorders framework and 2 assessing clinical significance, each rated on an 8-point Likert scale. For each item, a threshold was applied to convert the response into a binary score. For each diagnostic criterion, the relevant item scores were then summed; a summed score of 1 or greater was recoded as 1 (criterion met), whereas a summed score of 0 indicated that the criterion was not met. The number of criteria met across the 11 diagnostic criteria was summed to yield the symptom count, ranging from 0 to 11. When clinical symptoms are present, endorsing 2–3 criteria denotes a mild level, 4–5 a moderate level, and 6 or more a severe level of food addiction. Given this dichotomous scoring, reliability was estimated using the Kuder–Richardson 20 (KR-20) coefficient, which is appropriate for dichotomously scored items. The KR-20 reliability coefficient was .98 in the study by Shin et al. [35] and .91 in this study.

### 4. Data collection

Data were collected from May 25 to August 10, 2023. Participants were recruited through announcements posted on bulletin boards at Namseoul University. To minimize postprandial effects on appetite-related responses, measurements were scheduled during presumed preprandial time windows, either 11:00–12:00 or 16:00–18:00. Participants completed the assessment procedures in the following order: (1) provision of written informed consent, (2) body composition assessment using the InBody 970 device, (3) YOF test, and (4) self-administered questionnaires. The total duration of all assessments was approximately 30 min per participant. Both the InBody device and the YOF test kit are noninvasive and widely used in clinical practice, with no known physical, social, legal, or economic risks; participants were nonetheless informed that rest and olfactory recovery measures were available at any time. No adverse effects were reported. All participants received a small gift as compensation for their participation.

### 5. Data analysis

All statistical analyses were performed using SPSS version 24.0 (IBM Corp., Armonk, NY, USA) and the PROCESS macro version 4.2. In the primary correlation, regression, and mediation analyses, BMI and food addiction symptom scores were analyzed as continuous variables. BMI was maintained as a continuous outcome in the primary mediation analyses because converting continuous measures into categories can lead to information loss and decreased statistical power [36]. For clinical interpretability, supplementary analyses were also performed by categorizing participants as underweight/normal weight, overweight, or obese based on the aforementioned criteria [2]. Thus, the analytical approach was consistent with previous mediation studies of food addiction and BMI, in which BMI was treated as a continuous outcome [28,29]. Participants’ BMI, olfactory function, food addiction, and depression levels were analyzed using descriptive statistics. To analyze BMI differences across general characteristics, t-tests and analysis of variance were used, followed by Scheffé’s test for post-hoc analysis. Chi-square tests were used to evaluate the associations between BMI categories and the categorical classifications of olfactory function, depressive symptoms, and food addiction. For the categorical analyses, the underweight and normal-weight groups were combined because there were few underweight participants. The chi-square test assumption was met, as fewer than 20% of cells (16.7%) had expected frequencies below 5. Correlations among BMI, olfactory function, food addiction, and depression were analyzed using Pearson’s correlation coefficient. Multiple regression analysis was used to evaluate factors affecting BMI. Before conducting the regression analysis, the Durbin–Watson test was applied to assess residual autocorrelation, and the tolerance and variance inflation factor (VIF) were used to assess multicollinearity. Residual analysis was used to verify model linearity, normality of errors, and homogeneity of variance. The PROCESS macro (Model 4) was used with 10,000 bootstrap resamples to test the mediating effect of food addiction.

### 6. Ethical considerations

Prior to participant recruitment, the Institutional Review Board (IRB) of the authors’ institution reviewed and approved the study protocol (IRB No.: NSU-202304-001). After approval, participants were recruited through notices posted at the affiliated institution. Participation was entirely voluntary, and participants who voluntarily agreed to participate after reading the recruitment notices signed written informed consent forms before completing the tests and questionnaires. Before enrolling, each participant received an explanation of the study’s aims, procedures, potential risks and benefits, and confidentiality. Anonymity and confidentiality were maintained throughout the study. They were told that their responses would be used for research purposes only, held securely, and then destroyed in accordance with applicable regulations. They were also told that they could withdraw from the study at any time without penalty or disadvantage. No personal identifying details were collected, and all study-related data will be stored securely for 3 years following study completion and then permanently destroyed.

## RESULTS

### 1. BMI, olfactory function, depression, and food addiction

Table 1 summarizes participants’ BMI, olfactory function, depression, and food addiction. Participants’ mean BMI was 24.02 ± 3.87 kg/m^2^, and 66 participants (41.8%) were classified as underweight/normal weight (BMI < 23.0 kg/m^2^), 43 (27.2%) as overweight (23.0–24.9 kg/m^2^), and 49 (31.0%) as obese (≥ 25.0 kg/m^2^) (Table 2). The mean total score for olfactory function was 23.73 ± 3.16 points, whereas the mean scores for depression and food addiction were 4.32 ± 4.64 and 2.85 ± 2.48 points, respectively. All skewness and kurtosis values fell within the acceptable limits (|skewness| < 2, |kurtosis| < 7) for parametric analysis.

### 2. Differences in BMI according to general characteristics

Table 2 presents participants’ characteristics and BMI differences. Most participants were women (65.2%), aged ≤ 29 years (85.4%), lived in multi-person households (86.1%), were current drinkers (75.3%), and had never smoked (68.3%). Smoking status was the only characteristic significantly associated with BMI (F = 6.12, *p* = .003); current smokers had a higher BMI than former smokers.

### 3. Associations between BMI categories and olfactory function, depression, and food addiction

BMI categories were not significantly associated with olfactory function (χ² = 0.15, *p* = .927), depression (χ² = 3.01, *p* = .222), or food addiction (χ² = 4.81, *p* = .569) (Table 3).

### 4. Mediation of food addiction on the effects of olfactory function and depression on BMI

Correlations among the study variables were examined first. Food addiction was positively correlated with both olfactory function (r = .16, *p* = .047) and depression (r = .49, *p* < .001). However, BMI was not significantly correlated with olfactory function (r = .01, *p* = .918), depression (r = −.11, *p* = .191), or food addiction (r = .06, *p* = .441). Before testing mediation, regression assumptions were verified. The Durbin–Watson statistic was 2.21, indicating no evidence of autocorrelation, while VIF values ranged from 1.06 to 1.33, indicating no multicollinearity concerns. In the model with food addiction as the outcome, depression was a significant predictor (β = .49, *p* < .001), whereas olfactory function was not (β = .13, *p* = .076); this model accounted for 27.0% of the variance. In the model predicting BMI, smoking status was included as a covariate because it was significantly associated with BMI, with never smoking used as the reference category in the dummy coding. Current smoking (β = .22, *p* = .006), depression (β = −.20, *p* = .024), and food addiction (β = .18, *p* = .044) significantly predicted BMI, whereas olfactory function did not (β = −.02, *p* = .769). Overall, the model accounted for 11.0% of the variance in BMI (Table 4).

Next, the PROCESS macro was used to examine the mediating effect of food addiction in the associations between olfactory function and depression with BMI. Current smoking status was dummy-coded and controlled for, and olfactory function and depression were entered simultaneously as predictors so that each was adjusted for the other. The mediating effect’s statistical significance was tested using bootstrapping, and an effect was considered significant when the 95% confidence interval (CI) did not include zero.

First, in the test of the mediating effect of food addiction in the association between olfactory function and BMI (Table 4), neither the direct effect of olfactory function on BMI (B = −0.03, 95% CI: −0.22 to 0.16) nor the total effect was statistically significant (B = 0.00, 95% CI: −0.19 to 0.19). Finally, the indirect effect tested using the bias-corrected bootstrap was not statistically significant, as the 95% bias-corrected (BC) CI included zero (B = 0.03, 95% BC CI: −0.01 to 0.07); thus, food addiction did not significantly mediate the olfactory function–BMI relationship.

**Figure 1.**
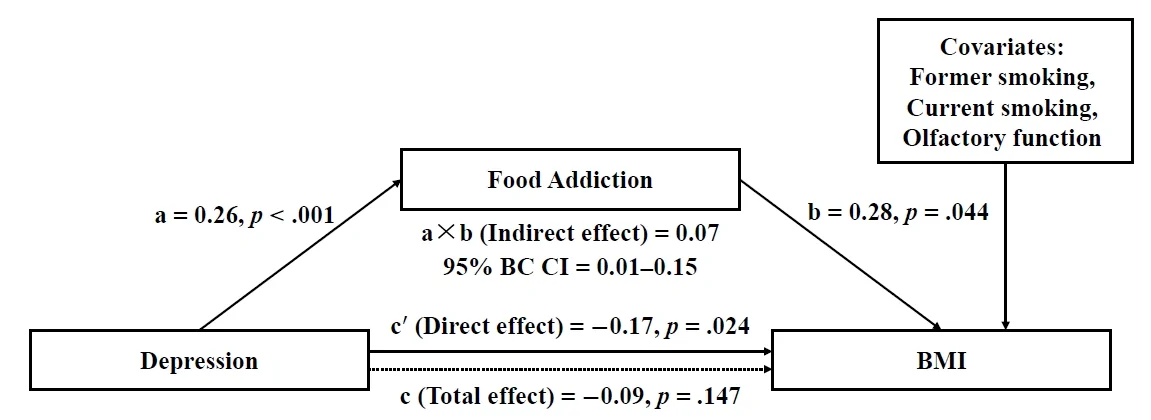
Mediation model of depression, food addiction, and BMI. a = direct effect of depression on food addiction; b and c′= direct effects of food addiction and depression on body mass index (BMI), respectively; c = total effect of depression on BMI; a×b = indirect effect of depression on BMI through food addiction; 95% BC bootstrap CI = 95% bias-corrected bootstrap confidence interval. BMI = Body mass index.

Second, in the test of the mediating effect of food addiction in the association between depression and BMI (Table 4, Figure 1), the direct effect of depression on BMI was significantly negative (B = −0.17, 95% CI: −0.31 to −0.02), whereas the total effect was not statistically significant (B = −0.09, 95% CI: −0.22 to 0.03). Finally, the indirect effect of depression on BMI through food addiction was statistically significant, as the 95% BC CI ranged from 0.01 to 0.15 and did not include zero (B = 0.07). That is, depression exerted a positive indirect effect on BMI through food addiction, whereas its direct effect was negative, indicating an inconsistent mediation (suppression) pattern in which the two effects operated in opposite directions.

## DISCUSSION

This study aimed to determine the effects of olfactory function and depression on BMI, with food addiction as a mediator, in young Korean adults. The results confirmed that food addiction mediated the depression–BMI relationship. Because the sample consisted predominantly of young adults, the findings are discussed primarily with reference to this age group.

Participants had a mean BMI of 24.02 ± 3.87 kg/m^2^, placing the sample average within the overweight category based on the aforementioned criteria [2]. In addition, 58.2% of participants were categorized as overweight or obese. As the sample was drawn from a university setting and had a mean age of 25.10 years, this profile aligns with national data showing rising obesity in Korea, most pronounced among adults in their 20s and, for class II–III obesity, among those in their 20s and 30s [4,30]. These findings suggest that excess body weight is already prevalent during young adulthood and underscore the importance of examining factors associated with BMI in this population.

Depression was significantly associated with food addiction, consistent with reports that depression significantly predicts food addiction in university students in Korea [24] and young adults in the U.S. [11]. One study reported it as a key correlate of food addiction, with affected individuals showing higher levels of depression and impulsivity [12]. Individuals with depressive symptoms often engage in emotional eating and develop addictive eating habits in response to stress and negative emotions [37]. Furthermore, depression is associated with psychological factors such as low self-esteem, body shape concerns, and impaired emotional regulation, which also increase food addiction risk [38]. Therefore, healthcare providers must recognize the association between depression and food addiction. In obesity prevention and treatment, understanding these relationships can inform the development of personalized interventions that address both mental health and maladaptive eating behaviors.

Although food addiction was not significantly correlated with BMI, it emerged as a significant positive predictor of BMI and significantly mediated the depression–BMI relationship. Food addiction involves intense cravings and difficulty controlling eating behavior. These behaviors tend to drive energy intake above what the body needs, leading to weight gain [10,25,39]. A meta-analysis indicated that food addiction is more prevalent among overweight and obese individuals, with the highest rates observed in those with the highest BMIs [12]. These findings align with evidence that emotional eating mediates the effect of depression on weight gain [37].

Previous studies have reported that depression is a psychological contributor to obesity, with affected individuals at greater risk than unaffected individuals [40–42]. In the present study, however, the positive association between depression and BMI occurred only indirectly through food addiction, whereas the direct effect of depression on BMI was negative. This inconsistent mediation pattern suggests that depression may influence body weight through two opposing pathways. One may involve reduced appetite and weight loss, as reflected in the negative direct effect. The other may involve coping with negative affect through addictive-like consumption of energy-dense food, as represented by the positive indirect effect through food addiction. This interpretation is supported by evidence that the mediating effect of food addiction on the relationship between depression and BMI is stronger among individuals with increased, rather than decreased, appetite [28]. The present findings are consistent with, while also extending, the results of two previous mediation studies. In a general adult sample, food addiction fully mediated the relationship between depressive symptom severity and BMI, with a stronger effect observed among participants reporting increased appetite [28]. Similarly, a study of young adults in Taiwan found that food addiction mediated the association between perceived stress and BMI [29]. The present study demonstrates a comparable mediating role of food addiction among young Korean adults but examines depression rather than perceived stress as the predictor. This finding suggests that the mediating pathway is not specific to a single form of negative affect. However, neither of the previous studies reported the suppression pattern identified in the present analysis. One plausible explanation may lie in differences between the affective constructs examined. Perceived stress [29] is less directly associated with appetite loss than depression, for which diagnostic criteria include both increased and decreased appetite. Further support for this interpretation comes from a recent cross-sectional study of adults, which found that higher BMI was associated with greater food addiction severity but not depressive symptom severity [43]. This dissociation closely resembles the correlation pattern observed in the present study. Neurobiological mechanisms may underlie both directions. Depression can disturb hypothalamic–pituitary–adrenal axis activity, driving cortisol overproduction and, in turn, weight gain [44]. It may also promote overeating and weight gain by altering neurotransmitters involved in appetite regulation and reward processing [25]. These findings have important practical implications. Although depressive symptoms may be difficult to resolve in the short term, addictive-like eating represents a behavioral pathway that can potentially be identified through screening and targeted for modification. Food addiction may therefore represent a clinically relevant and modifiable intervention target for reducing the risk of weight gain among young adults with depressive symptoms. Interventions focusing solely on depression may be less effective for weight management unless co-occurring maladaptive eating behaviors are addressed simultaneously.

Olfactory function was not significantly associated with BMI in this study. Previous studies on the olfactory function–obesity relationship have reported inconsistent results. For example, one study reported reduced olfactory perception with increasing body weight [15], while another reported higher olfactory sensitivity in individuals with high BMI compared to those of healthy weight [21]. Two characteristics of the present sample may further account for the null finding. First, olfactory function was generally well preserved among this young adult sample. The mean total olfactory function score was 23.73 ± 3.16, exceeding the normal cut-off of 22 points, and only 27.8% of participants were classified as having olfactory dysfunction. Normative evidence suggests that olfactory performance peaks and varies relatively little between 20 and 30 years of age [45]. Consequently, the restricted variability in olfactory scores within a young adult sample may limit the statistical power available to identify an association with BMI. Consistent with this interpretation, a study of patients attending an obesity center found a significant negative correlation between olfactory scores and BMI among overweight and obese patients but none among normal-weight controls [46]. Second, olfactory function showed a weak unadjusted correlation with food addiction symptoms (r = .16, *p* = .047), but the association was no longer statistically significant after adjustment. Therefore, this finding should be interpreted as exploratory. Although the direction of the association is consistent with the reward-related pathway proposed in the present study, the data do not provide confirmatory support for it. Future research, including participants with greater variability in olfactory function, may provide a stronger basis for evaluating it.

Among the covariates examined, current smoking demonstrated the strongest association with BMI. Obesity arises from complex interactions between genetic and lifestyle factors, with smoking identified as a key behavioral contributor [40] and an established risk factor [41]. Among young people, lower nicotine dependence and weaker craving have been identified as probable predictors of smoking abstinence [47], suggesting that addressing dependence may be a useful component of cessation support in this age group. Accordingly, smoking status may be an important factor to consider when assessing weight-management needs among young adults.

When BMI was examined as a categorical variable, no significant associations were observed with olfactory function, depression, or food addiction. These supplementary analyses were performed to facilitate clinical interpretation based on established BMI cut-offs. However, converting a continuous variable into categories can result in information loss and reduced statistical power [36], a concern that is especially relevant here because of the modest associations in the continuous analyses (e.g., r = −.11 for depression and an indirect effect of B = 0.07). In addition, categorizing BMI removes individual differences so that the associations that actually exist may appear attenuated, particularly for values near the boundary between normal weight and overweight. Distributing 158 participants across as many as 12 cells, with only 17 in the depressed range, left some category combinations with few participants. Thus, BMI was retained as a continuous outcome in the primary analyses, and the nonsignificant categorical findings should be interpreted as reflecting the reduced sensitivity of categorical analyses in a sample of this size rather than a true absence of association. Overall, the present results should be regarded as preliminary evidence of potentially modifiable weight-related pathways in young adulthood rather than as diagnostic evidence concerning clinical obesity.

This study offers several implications for biological nursing science. By integrating objective physiological measures with psychological assessments of depression and food addiction, it demonstrates a biopsychosocial approach in which psychological and physiological dimensions of health are examined together. Although olfactory function was not significant, the use of a standardized sensory-physiological measure provides a methodological reference for future nursing research on sensory and eating-related physiological factors. Future research could combine olfactory testing with measures of appetite-regulating hormones to better identify the conditions under which olfactory function and BMI are associated.

The mediating role of food addiction between depression and BMI also has clinical relevance. Nurses caring for young adults at risk of excess weight gain or higher BMI may consider evaluating addictive-like eating behaviors together with depressive symptoms, particularly in primary care and community health settings. In clinical practice, depression screening could be accompanied by food addiction assessment among young adults who report depressive symptoms [28]. Targeted interventions, particularly mindfulness-based strategies aimed at reducing craving, could then be considered alongside appropriate referrals to mental health professionals [48]. As food addiction, rather than depressive symptoms alone, was associated with higher BMI in this sample, nursing interventions may need to address eating behavior explicitly rather than relying solely on mental health support. Weight-management programs for young adults may also benefit from integrating dietary and psychological approaches, including whole-food diets, cognitive behavioral therapy, and mindful eating [49]. Establishing collaborative networks among nurses, mental health professionals, nutritionists, and exercise specialists may further support a coordinated approach to addressing depression, food addiction, and obesity. In addition, healthcare provider training should incorporate the screening and management of depression and addictive-like eating to reduce the likelihood of overlooking this potentially important behavioral pathway in routine clinical care.

Several limitations should be considered when interpreting these results. First, participants were recruited from a single university, and their student or staff status was not recorded, potentially limiting the representativeness and generalizability of the sample to broader young-adult populations. In addition, although recruitment targeted young adults, no upper age limit was applied, and 16 participants (10.1%) were older than 39 years. The findings should therefore be interpreted as applying primarily to young adults, and replication in samples restricted to this age range is warranted. Second, the cross-sectional design precluded assessment of causal relationships, including that between smoking status and BMI. Longitudinal studies are needed to clarify causality and identify potential mediators or moderators. Third, most participants had olfactory function scores within the normal range, which may have weakened the observed association between olfactory function and BMI. Fourth, the categorical BMI analyses had limited statistical power because some cells contained relatively few participants; therefore, the null results should be interpreted with caution. Finally, although assessments were scheduled for a preprandial window, participants’ actual time since the last meal and individual fasting status were not verified, and these factors may have influenced olfactory and appetite-related measures.

## CONCLUSION

This descriptive cross-sectional study investigated whether food addiction mediates the associations of olfactory function and depression with BMI among young Korean adults. In the sample of 158 adults, current smoking status, depression, and food addiction emerged as significant predictors of BMI, whereas olfactory function did not. Food addiction significantly mediated the relationship between depression and BMI. Notably, depression exerted a positive indirect association with BMI through food addiction, while its direct association with BMI was negative. These findings indicate that addictive-like eating behaviors may represent an important behavioral target for addressing higher BMI among young adults experiencing depressive symptoms.

Based on our findings, we offer the following recommendations. First, longitudinal studies are needed to clarify the temporal sequence among depression, food addiction, and weight gain and determine whether addictive-like eating behaviors precede increases in BMI during young adulthood. Second, future studies should directly assess appetite change or depressive subtypes to examine the opposing pathways underlying the suppression effect identified in the present study. Third, studies involving participants with a broader range of olfactory function, as well as those combining olfactory assessments with appetite-regulating hormones, are warranted to determine whether and under what conditions olfactory function is associated with BMI or weight change. Fourth, integrative intervention programs that address both depressive symptoms and addictive-like eating behaviors should be developed and validated among young adults. Such efforts may strengthen obesity prevention and management by enhancing mental health and dietary behaviors in this population.

## Figures and Tables

**Table 1.** Participants’ BMI, Olfactory Function, Depression, and Food Addiction Levels (N = 158)

Characteristics	Range	M ± SD	Min	Max	Skewness	Kurtosis
BMI (kg/m^2^)	-	24.02 ± 3.87	17.82	37.90	0.99	1.23
Olfactory function	0–36	23.73 ± 3.16	15	31	−0.03	−0.41
Depression	0–27	4.32 ± 4.64	0	25	1.94	4.38
Food addiction	0–11	2.85 ± 2.48	0	11	1.14	0.95

BMI = Body mass index; M = Mean; SD = Standard deviation; Min = Minimum; Max = Maximum.

**Table 2.** Differences in BMI According to Participants’ General Characteristics (N = 158)

Characteristics	Categories	n	%	BMI	t/F (*p*)
				M ± SD	Scheffé’s test
BMI (kg/m^2^)	Underweight/normal weight^†^ (< 23.0)	66	41.8	-	-
	Overweight (23.0–24.9)	43	27.2	-	
	Obese (≥ 25.0)	49	31.0	-	
Sex	Men	55	34.8	24.15±2.78	0.36 (.717)
	Women	103	65.2	23.95±4.36	
Age (years)	25.10 ± 9.37				
	≤ 29	135	85.4	23.92±3.82	−0.80 (.426)
	> 29	23	14.6	24.61±4.23	
Household type	Single-person	22	13.9	24.53±3.33	0.67 (.504)
	Multi-person	136	86.1	23.93±3.96	
Average sleep duration (hours)	< 6	42	26.6	24.29±4.35	0.85 (.469)
	6–7	59	37.3	23.58±3.27	
	7–8	35	22.2	24.74±4.12	
	> 8	22	13.9	23.53±4.03	
Smoking status	Never smoker^a^	108	68.3	23.90±4.04	6.12 (.003)
	Former smoker^b^	21	13.3	22.11±2.46	b < c
	Current smoker^c^	29	18.4	25.82±3.35	
Alcohol consumption	Nondrinker	28	17.7	24.47±4.63	2.11 (.125)
	Former drinker	11	7.0	21.77±2.64	
	Current drinker	119	75.3	24.12±3.73	
Exercise type	Very light physical activity	23	14.6	23.57±4.17	1.01 (.390)
	Walking	72	45.5	24.07±4.23	
	Moderate physical activity	35	22.2	23.41±3.75	
	Vigorous physical activity	28	17.7	25.01±2.59	
Exercise frequency	Rarely	42	26.6	23.28±4.26	1.27 (.287)
	1–2 times/week	44	27.8	24.11±4.19	
	3–4 times/week	42	26.6	23.91±3.14	
	≥ 5 times/week	30	19.0	25.07±3.69	
Stress level	None	6	3.8	23.76±3.67	0.93 (.448)
	Occasionally	59	37.3	23.93±3.45	
	Sometimes	58	36.7	24.07±3.85	
	Often	24	15.2	24.96±4.43	
	Always	11	7.0	22.27±4.94	

BMI = Body mass index; M = Mean; SD = Standard deviation.

† Underweight, < 18.5 kg/m^2^; normal weight, 18.5–22.9 kg/m^2^.

**Table 3.** Associations between BMI Categories and Olfactory Function, Depression, and Food Addiction (N = 158)

Variables	Categories	BMI						
		Underweight/normal weight^†^ (< 23.0 kg/m^2^)		Overweight (23.0–24.9 kg/m^2^)		Obese (≥ 25.0 kg/m^2^)		χ^2^ (*p*)
		n	%	n	%	n	%	
Olfactory function	Normal (≥ 22)	47	41.2	32	28.1	35	30.7	0.15 (.927)
	Dysfunction (≤ 21)^‡^	19	43.2	11	25.0	14	31.8	
Depression	Absent (< 10)	56	39.7	41	29.1	44	31.2	3.01 (.222)
	Present (≥ 10)	10	58.8	2	11.8	5	29.4	
Food addiction	Normal	21	36.9	19	33.3	17	29.8	4.81 (.569)
	Mild	26	51.0	10	19.6	15	29.4	
	Moderate	8	30.8	8	30.8	10	38.4	
	Severe	11	45.8	6	25.0	7	29.2	

BMI = Body mass index.

† Underweight, < 18.5 kg/m^2^; normal weight, 18.5–22.9 kg/m^2^;

‡ Olfactory dysfunction includes hyposmia and anosmia.

**Table 4.** Mediating Effect of Food Addiction in the Associations of Olfactory Function and Depression with BMI (N = 158)

Variables	Food addiction						BMI				
	B	SE	β	*p*			B		SE	β	*p*
Constant	−0.44	1.35		.744			24.44		2.33		< .001
Former smoking^†^	−0.23	0.52	−.03	.662			−1.74		0.90	−.15	.055
Current smoking^†^	−0.78	0.45	−.12	.085			2.18		0.79	.22	.006
Olfactory function	0.10	0.06	.13	.076			−0.03		0.10	−.02	.769
Depression	0.26	0.04	.49	< .001			−0.17		0.07	−.20	.024
Food addiction							0.28		0.14	.18	.044
R^2^	.27					.11					
F (*p*)	14.18 (< .001)					3.76 (.003)					
Path					B			SE		95% CI	
Total effect											
Olfactory function → BMI					0.00			0.10		−0.19 to 0.19	
Depression → BMI					−0.09			0.07		−0.22 to 0.03	
Direct effect											
Olfactory function → BMI					−0.03			0.10		−0.22 to 0.16	
Depression → BMI					−0.17			0.07		−0.31 to −0.02	
Indirect effect											
Olfactory function → Food addiction → BMI					0.03			0.02		−0.01 to 0.07	
Depression → Food addiction → BMI					0.07			0.04		0.01–0.15	

Coefficients are rounded to two decimal places; therefore, the total effect may not exactly equal the sum of the direct and indirect effects. Indirect effects were tested with 95% bias-corrected bootstrap confidence intervals based on 10,000 resamples. VIF = 1.06–1.33; Durbin–Watson = 2.21. BMI = Body mass index; SE = Standard error; CI = Confidence interval.

† Smoking status was dummy-coded, with never smokers as the reference category.
